# Gastroenteropancreatic Neuroendocrine Neoplasms: Clinical and Translational Perspectives

**DOI:** 10.31662/jmaj.2026-0243

**Published:** 2026-08-07

**Authors:** Kenta Kawasaki

**Affiliations:** 1 Division of Gastroenterology and Hepatology, Department of Internal Medicine, Keio University School of Medicine, Tokyo, Japan; 2 Keio Cancer Center, Keio University School of Medicine, Tokyo, Japan

**Keywords:** GEP-NET, GEP-NEC, organoid, NETG3, Ki-67

## Abstract

Gastroenteropancreatic neuroendocrine neoplasms (GEP-NENs) are a heterogeneous group of malignancies characterized by the expression of neuroendocrine markers. In recent years, GEP-NENs have gained increasing clinical attention because of their rising prevalence and the need for more effective therapeutic strategies for them. GEP-NENs are classified into gastroenteropancreatic neuroendocrine tumors (GEP-NETs) and gastroenteropancreatic neuroendocrine carcinomas (GEP-NECs), based on the Ki-67 proliferation index and histological differentiation. GEP-NETs are generally characterized by relatively indolent clinical behavior, typically limited genomic alterations, and responsiveness to molecularly targeted therapies, whereas GEP-NECs are highly aggressive neoplasms frequently harboring *TP53* and *RB1* alterations, and are typically treated with cytotoxic chemotherapy. Though these definitions provide a useful framework for tumor classification, real-world GEP-NENs frequently exhibit substantial intra- and intertumoral heterogeneity, making clinical interpretation considerably more complex. Recently, a subset of well-differentiated neoplasms with high Ki-67 indices has been recognized as gastroenteropancreatic neuroendocrine tumors G3 (GEP-NETG3), representing tumors that do not fit neatly into the conventional classification system. Treatment strategy for GEP-NETG3 highlights the difficulties faced for filling the gap between pathological classification and biomarkers currently used to guide clinical management. Addressing this challenge requires a multifaceted approach, integrating clinical and translational research to better capture the biological complexity of these tumors.

In this review, we summarize the current understanding of GEP-NENs, focusing on their biological features, therapeutic implications, and emerging insights from both clinical and translational research perspectives.

## Introduction

Cancer comprises a heterogeneous group of diseases characterized by uncontrolled proliferation, invasion, and metastatic potential ^(1)^. Despite remarkable advances in cancer genomics and therapeutics, the biological behavior of tumors remains incompletely understood. This complexity arises from multiple layers of heterogeneity, including genetic alterations, epigenetic states, and tumor microenvironmental influences. One of the unique phenotypes among this heterogeneity is the histological feature. Each tumor has its unique aspect that form intra- and intertumor heterogeneity, yet the mechanisms by which such diversity is established and maintained remain poorly understood. Recent understanding in stem cell biology gave us the insight that cancer originates from the original tissue stem cells ^(2)^. This concept is in line with the observation that tumor histology generally mirrors the epithelial lineage of the tissue of origin. For instance, gastric and colorectal cancers are predominantly adenocarcinomas derived from glandular epithelium, whereas esophageal and anal cancers are more commonly squamous cell carcinomas originating from squamous epithelium ^(3)^. In this context, it is particularly interesting that a distinct tumor entity known as neuroendocrine neoplasm (NEN) exists. NENs are characterized by the expression of neuroendocrine markers such as synaptophysin, chromogranin A, and *NCAM1* (CD56), and can occur in various organs throughout the body ^(4)^. Although these criteria fulfill the fundamental characteristics of neuroendocrine cells, their cellular origin is still unknown and controversial.

NENs are heterogenous diseases, and they are categorized into two broad disease categories, namely, well-differentiated neuroendocrine tumors (NETs) and poorly differentiated neuroendocrine carcinomas (NECs) ^(4)^. Although these two entities are included in the same terminology, the prognoses for newly diagnosed NETs and NECs are completely different. NETs are typically slow-progressing tumors and are often asymptomatic. Despite their slow-progressing phenotype, NETs can metastasize, and a higher tumor grade is associated with an increased risk of metastasis. Based on the slow-progressing nature of the tumors, overall survival is measured in years even after metastatic disease is diagnosed. In contrast, NECs are highly aggressive neoplasms characterized by rapid proliferation and a frequent metastatic potential, with a median survival of only 2-3 months in the absence of treatment. Approximately 70% of patients present with metastatic disease at diagnosis ^(5)^. Although NETs and NECs differ markedly in prognosis and clinical course, accurate distinction between the two entities can be challenging in certain cases. Based on these cases, the World Health Organization (WHO) classification provided a new classification named as neuroendocrine tumors G3 (NETG3) in 2017 ^(6)^.

This terminology covers NEC cases with characteristics of NETs, including poor responsiveness to chemotherapy. Although NETG3 is a unique entity for gastroenteropancreatic NEN (GEP-NEN), understanding these borderline cases might provide a new avenue for elucidating the biology of this disease. In this review, we focus on NENs of the digestive tract, which are termed gastroenteropancreatic NETs (GEP-NETs) and gastroenteropancreatic NECs (GEP-NECs), to discuss our current knowledge of clinical and translational aspects of these diseases.

## Classification of NEN

NETs were first reported in 1907 by Siegfried Oberndorfer under the name “carcinoid” ^(7)^. As indicated from the name of this disease “Karzinoide (carcinoma-like),” initially, NETs were considered as benign and not malignant ^(8)^. This impression was caused by its histology, which has less nuclear atypia and well-differentiated feature. However, in 1928, this was revised according to their metastatic potential ^(9)^. At that time, people learned that there are NETs that have more aggressive potential. First described in the 1920s, this tumor was called as “Oat cell carcinoma” based on its morphology ^(10)^. This is the currently called “small cell lung cancer (SCLC).” The recognition of these two disease entities formed the basis of the WHO classification in 2000 ^(11), (12)^. After the establishment of this classification, it was revised in 2004, 2010, 2017, 2019, and most recently in 2022 ^(4), (6), (13), (14)^. These classifications are updated periodically to incorporate new biological insight.

The current diagnosis of GEP-NENs is based on morphologic characteristics supported by immunohistochemistry (IHC) staining for neuroendocrine markers such as chromogranin A, synaptophysin, and CD56. Most GEP-NETs express these neuroendocrine markers, while their expression in GEP-NECs is variable and heterogeneous. Assessment of morphological differentiation, mitotic frequency, and Ki-67 proliferation rate of the tumor serve as the basis of pathologic diagnosis. In the current pathological classification, GEP-NETs are divided into three categories: NETG1, G2, and G3 (Table 1). NETG1 has less than 2 mitoses/2 mm^2^, G2 has 2-20 mitoses/2 mm^2^, and G3 has more than 20 mitoses/2 mm^2^ (Figure 1A and B)^(6)^. The recently designated GEP-NETG3 (WHO classification 2017), was based on the observation that GEP-NETs demonstrate less response to chemotherapy and a higher proliferative index (up to Ki-67 index of 55%) compared to typical GEP-NETs, but retain a well-differentiated morphology relative to GEP-NECs ^(15)^. Tumor differentiation is important for distinguishing between GEP-NETG3 and GEP-NEC, and IHC staining of *TP53*, *RB1*, and somatostatin receptors (SSTR) can be useful.

**Table 1.** Characteristics of GEP-NETG1, G2, and G3, as well as GEP-NEC.

	GEP-NET			GEP-NEC	
Pathological classification	Grade 1	Grade 2	Grade 3	Small-type	Large-type
Differentiation	Well-differentiated			Poorly differentiated	
Ki-67 index (%)	<3	3-20	20<	20<	
Clinical characteristics	Usually indolent		Slightly aggressive	Aggressive, highly metastatic	
*TP53, RB1* status	Maintained			Lost	

GEP-NEC: gastroenteropancreatic neuroendocrine carcinoma; GEP-NET: gastroenteropancreatic neuroendocrine tumor; GEP-NETG1: gastroenteropancreatic neuroendocrine tumor G1.

**Figure 1.**
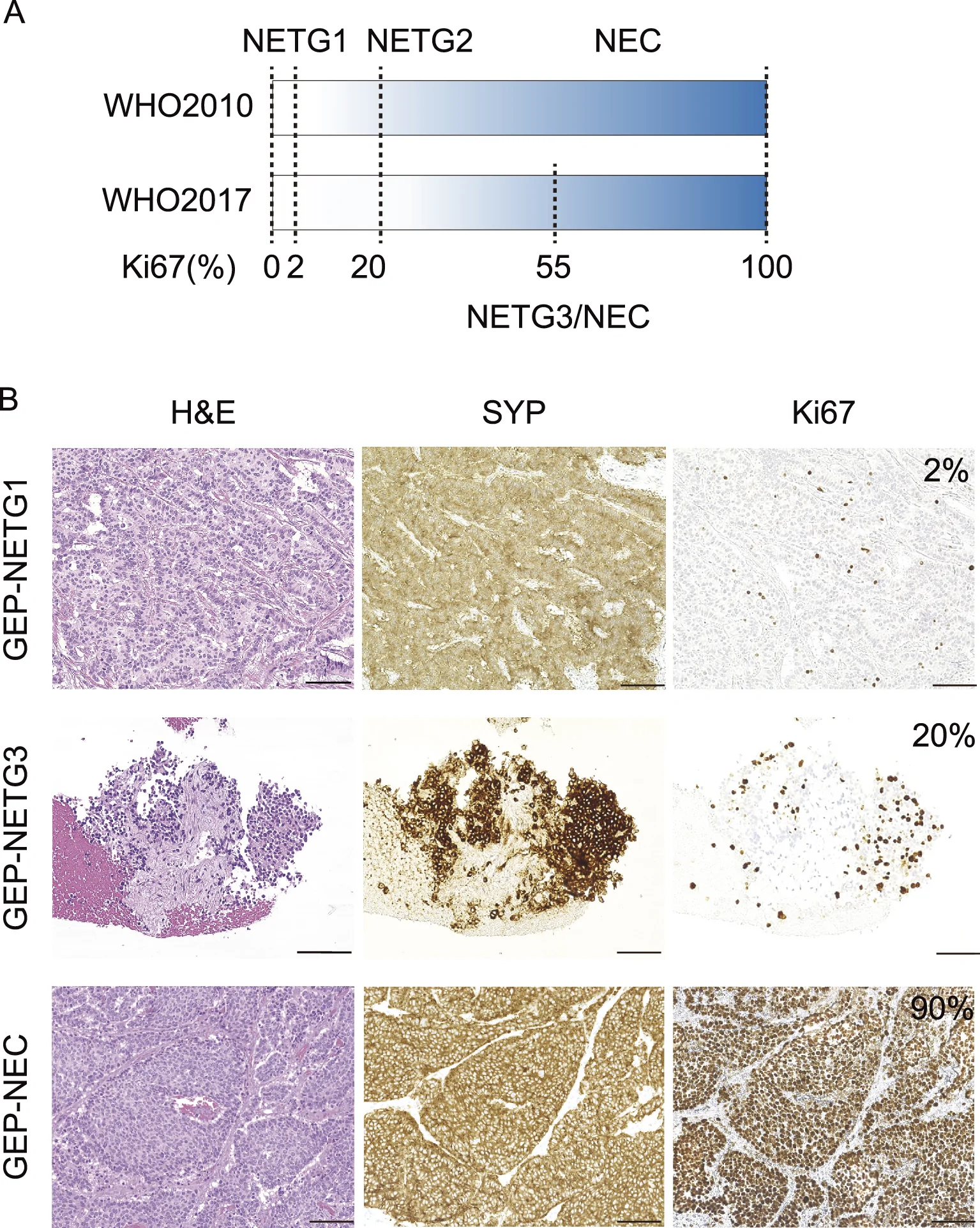
Classification of NEN. (A) World Health Organization (WHO) classification 2010 and 2017 of GEP-NEN, which is classified by Ki-67 index. In the WHO classification 2017, Ki-67 index above 20% are classified as either NETG3 (well-differentiated) or NEC (poorly differentiated). A Ki-67 index of 55% is one of the useful landmarks to distinguish NETG3. (B) Representative histology of GEP-NETG1, G2, and G3, as well as GEP-NEC. Hematoxylin and eosin (H&E), synaptophysin (SYP), and Ki-67 staining are shown. Ki-67 labeling indices are indicated in each panel. Scale bar: 100 μm. GEP-NEN: gastroenteropancreatic neuroendocrine neoplasm; GEP-NET: gastroenteropancreatic neuroendocrine tumor; NEC: neuroendocrine carcinoma; NEN: neuroendocrine neoplasm.

GEP-NECs are divided into two histological subtypes namely, small cell and large cell, based on the size of the cells. Their prognosis is different in the lungs (large cell NEC and SCLC), but the clinical significance in GEP-NECs is unknown due to its limited number of cases.

There are some clinical cases that have combined histology of non-neuroendocrine and neuroendocrine components. Regardless of whether they are GEP-NETs or GEP-NECs, these tumors with mixed histological subtypes are designated as mixed neuroendocrine–non-neuroendocrine neoplasm (MiNEN), with each component comprising more than 30% of the tumor.

Although these definitions provide a useful framework for classifying tumors into distinct phenotypic entities, real-world GEP-NENs frequently exhibit substantial intra- and intertumoral heterogeneity, making clinical interpretation considerably more complex. Indeed, existence of entities such as GEP-NETG3, which lies between GEP-NETs and GEP-NECs, as well as MiNENs, highlight the limitations of the current classification system. Furthermore, these histological subtypes are often difficult to diagnose accurately based on a single biopsy specimen that may not fully represent the overall tumor landscape. For example, the Ki-67 index can vary not only among different tumors but also across distinct regions within the same tumor. In addition, proliferative activity of a cancer cell may change over time or be altered under therapeutic pressure. Therefore, a deeper biological understanding of GEP-NENs is urgently needed to improve prognostic stratification and to optimize prediction of treatment response in clinical practice.

## Biological Understanding of GEP-NEN

It is important to understand the genomic mutation profiles to understand the biological heterogeneity of GEP-NEN. Although familiar NETs have been recognized for many years, the genetic landscape of sporadic GEP-NETs and GEP-NECs remained unknown until recently.

GEP-NETs can be broadly classified into those arising in the setting of familial syndromes and those occurring sporadically. Approximately 10%-20% of GEP-NETs are associated with familial syndromes, including multiple endocrine neoplasia type 1 (MEN1), von Hippel–Lindau (VHL) disease, tuberous sclerosis complex, and neurofibromatosis type 1. In contrast, most sporadic GEP-NETs lack recurrent, clearly defined driver mutations. However, the overlap of genetic alterations between familial and sporadic GEP-NETs highlights the central role of cell cycle dysregulation in the development of GEP-NETs. Notably, alterations in *MEN1* are observed in both familial cases and in approximately 30% of sporadic pancreatic NETs ^(16)^. Though *MEN1* mutations are less frequent in small intestinal NETs, mutations in *CDKN1B*, which functionally interacts with *MEN1* in regulating the cell cycle, have been reported in around 10% of cases ^(16), (17)^. In addition, pancreatic NETs frequently harbor mutations in *DAXX* (44%) and *ATRX* (43%), both of which are involved in the maintenance of the telomere length ^(16)^.

In contrast to GEP-NETs, no familial syndromes of GEP-NEC have been reported to date. These tumors are characterized by frequent inactivation of the cell cycle regulators *TP53* and *RB1*, which is observed across multiple organs ^(18)^. In certain organs, such as the rectum and larynx, viral infections may contribute to the loss of both genes ^(19), (20)^. Although high frequency of *TP53* and *RB1* inactivation underscores their critical role in NEC development, loss of these tumor suppressors alone is insufficient for NEC tumorigenesis ^(21)^. Additional genomic alterations are often organ-specific and resemble those found in conventional adenocarcinomas arising from the same anatomical sites, including *APC*, *KRAS*, and *BRAF* mutations, as well as *EML4–NTRK3* fusions in colorectal NECs, *KRAS* mutations in pancreatic NECs, and *ELF3* mutations in ampullary and biliary NECs ^(18), (21)^. These key driver mutations are frequently shared between the neuroendocrine and non-neuroendocrine components of MiNENs, which suggest that they likely contribute primarily to tissue-specific tumorigenesis rather than directly driving neuroendocrine differentiation per se ^(22), (23)^. Indeed, introduction of these canonical driver mutations into normal colonic epithelium is still insufficient to recapitulate the NEC phenotype without concomitant activation of lineage-defining transcription factors ^(21)^. These findings suggest that while genetic alterations contribute to tumorigenesis, establishment of the neuroendocrine phenotype is governed primarily by transcriptional programs rather than by genomic alterations alone.

As expected from their distinct clinical behaviors, the genomic landscapes of GEP-NETs and GEP-NECs are markedly different. In low-grade GEP-NETs, inactivating mutations of *TP53* and *RB1* are exceedingly rare (less than 1%), whereas these alterations are present in approximately 80%-90% of GEP-NECs. An important question is how GEP-NETG3, which lies at the interface between GEP-NETs and GEP-NECs, fits into this framework. Recent studies have shown that a subset of GEP-NETG3 tumors harbors mutations associated with familial syndromes, such as MEN1 and VHL ^(24)^. In contrast, *TP53* and *RB1* alterations are less frequent than in GEP-NECs. In a large cohort of 465 NENs across multiple organs, normal p53 and retinoblastoma (RB) protein expressions were observed in 96.7% of NETG1/G2, 76.7% of NETG3, and only 5.8% of NECs. Conversely, abnormal p53 expression was detected in 12.7% of NETG3 and 91.5% of NECs, while RB loss was observed in 10.6% of NETG3 and 68.3% of NECs ^(25)^. These data indicate that GEP-NETG3 indeed carries genomically GEP-NET phenotype. Certain driver mutations, compared to classic GEP-NET, may contribute to the increased proliferative activity, as reflected by the elevated Ki-67 index. Although these findings arise from a different organ system and therefore cannot be directly extrapolated to GEP-NENs, it is noteworthy that evidence supporting a sequential relationship between NET and NEC has been reported in pulmonary NET. In a subset of pulmonary NETs, acquisition of chromothripsis has been associated with transformation into SCLC, a highly aggressive form of pulmonary NEC ^(26)^. These observations provide important insights into the plasticity and evolutionary trajectories of NENs.

## Treatment Strategy of GEP-NEN

Accurate diagnosis based on the histological criteria, supported by genomic mutation status, is important to consider the treatment strategy, since the standard treatment regimens differ substantially between GEP-NETs and GEP-NECs. Based on the slow proliferation, surgery is typically the first management consideration for resectable GEP-NETs. For unresectable GEP-NETs, there are several systemic therapies that have shown their efficacy. Based on their molecular characteristics, the SSTR axis and the mechanistic target of rapamycin (mTOR) signaling pathway represent major therapeutic targets in GEP-NETs. These pathways play central roles in the regulation of neuroendocrine cell proliferation and tumor growth. Somatostatin signaling influences NET proliferation through the activation of the mitogen-activated protein kinase (MAPK)/extracellular signal-regulated kinase (ERK) pathway ^(27)^. In particular, SSTR2 and SSTR5 are expressed on the cell surface of approximately 80% of NETs, and are considered key regulators of proliferation of the neuroendocrine cells ^(28)^. Most straightforward approach to this target is to block the receptor by somatostatin analogs (SSA) (octreotide and lanreotide), which have showed promising effects in previous clinical trials ^(29), (30)^. Recently, peptide receptor radionuclide therapy (PRRT), a novel radioligand-based therapeutic approach, has significantly advanced the treatment landscape for GEP-NETs. In this strategy, Lutetium-177-DOTA-Tyr3-octreotate (177Lu-DOTATATE) delivers targeted radiation to tumor cells by binding to SSTRs via a high-affinity SSA labeled with lutetium-177, a potent β-emitting radionuclide. Prior to treatment, the required evaluation of SSTR expression is typically performed using somatostatin receptor scintigraphy (SRS). Among the currently available therapies for GEP-NETs, 177Lu-DOTATATE has demonstrated one of the highest objective response rates in clinical trials, with response rates of approximately 15% and 45% reported in the NETTER-1 and NETTER-2 studies, respectively ^(31), (32)^. For alternative treatments, everolimus selectively targets mTOR, while sunitinib is a multi-kinase inhibitor targeting vascular endothelial growth factor receptor and platelet-derived growth factor receptor ^(33), (34), (35)^. Recently, large-scale clinical trial result of Cabozantinib is added to the repertoire of treatment strategy for GEP-NET ^(36)^. Although it is very limited, there is also evidence for classical cytotoxic chemotherapy used for GEP-NETs such as streptozocin regimen and capecitabine and temozolomide regimen ^(37), (38), (39)^. Their efficacy varies among patients.

In contrast to GEP-NETs, systemic cytotoxic therapy remains the cornerstone of treatment for GEP-NECs. This difference reflects the highly proliferative nature of GEP-NECs and their clinical resemblance to SCLC. Because of the rarity of GEP-NECs, prospective clinical trials have historically been difficult to conduct, and treatment strategies have largely been extrapolated from SCLC. Indeed, recent demonstration that platinum-etoposide and platinum-irinotecan remain appropriate first-line options for GEP-NECs suggests that NECs arising from different organs may share common therapeutic vulnerabilities ^(5), (40)^. Likewise, the success of immune checkpoint inhibitors and delta-like3 (DLL3)-targeted therapies in SCLC has generated considerable interest in extending these approaches to GEP-NECs ^(41), (42)^. However, an important question remains: to what extent are NECs arising from different organs biologically similar, and should they therefore be treated in the same manner? As discussed above, GEP-NECs harbor both pan-NEC genomic alterations, such as *TP53* and *RB1* inactivation, and organ-specific genomic alterations that resemble those found in conventional adenocarcinomas of the corresponding organs ^(5)^. This duality may have important therapeutic implications, as organ-specific genomic alterations may reveal actionable therapeutic targets beyond those addressed by conventional NEC therapies. Therefore, comprehensive genomic profiling (CGP) may provide an opportunity to identify treatment targets that would not be captured by a histology-driven strategy. Although large-scale CGP studies of GEP-NENs remain limited due to their rarity and heterogeneity, a study reports that 48% of patients with NENs (from all organ sites) harbored potentially actionable molecular alterations, of whom 35% subsequently received matched targeted therapies. As discussed, GEP-NECs harbored a broader spectrum of genomic alterations, increasing the likelihood of identifying therapeutically actionable events and expanding opportunities for precision medicine approaches. Although evidence remains limited, clinical activity of matched targeted therapies has been reported in patients and in patient-derived organoid (PDO) models harboring *BRAFV600E* mutations or *EML4–NTRK3* fusions ^(21), (43)^. On the contrary, most actionable alterations in NETs were from the mTOR pathway, which is already an established therapeutic target. Therefore, the additional clinical utility of CGP may be relatively limited in many patients with NETs.

Taken together, these findings support the use of CGP in patients with GEP-NEC, particularly after failure of standard therapies, where its clinical utility may be comparable to that observed in other advanced solid tumors. However, because patients with GEP-NEC often experience rapid clinical deterioration, performing CGP only after exhaustion of standard treatment options may not always allow sufficient time for genotype-matched therapeutic intervention. Therefore, the optimal timing of CGP testing warrants further investigation.

## Animal Models of GEP-NEN

To deepen our understanding of the biology of GEP-NENs and to facilitate new target identification, appropriate animal models of GEP-NETs and GEP-NECs are essential. Researchers tried to recapitulate the disease models by utilizing many animal models, such as genetically engineered mouse model, cell lines, patient-derived xenograft, rodent models, and zebrafish. Despite these efforts, very limited models of GEP-NETs and GEP-NECs exist ^(44)^. This is in part attributable to the rarity of the disease and its unknown biology. To overcome the limited availability of biologically relevant models for GEP-NETs and GEP-NECs, organoid technology has emerged as a promising experimental platform. Organoids are three-dimensional stem cell–derived culture systems that recapitulate key structural, genetic, and phenotypic features of the original tissue. Since their initial development, organoid cultures have been successfully established from a wide range of normal and malignant human tissues. The long-term maintenance of organoids is achieved by recreating the stem cell niche *ex vivo* through the use of an extracellular matrix scaffold, such as Matrigel, together with tissue-specific growth factors and signaling modulators. These commonly include Wnt/R-spondin signaling activators, the bone morphogenetic protein (BMP) inhibitor Noggin, epidermal growth factor, and other niche factors that support self-renewal of the tissue stem cells. As a result, organoids can maintain both genetic stability and key phenotypic characteristics of the original tissue over extended periods of culture ^(45), (46), (47)^. Importantly, organoids can be established not only from normal tissues but also from patient tumors, enabling the generation of PDOs across multiple organs, with success rates generally exceeding those of conventional cell line models ^(48)^. Given the rarity of the disease and the limited availability of human material in GEP-NEN, this approach represented a particularly suitable experimental platform. An organoid culture model for GEP-NEN has been developed and expanded to at least 25 GEP-NEN lines, including GEP-NEC, one gastrinoma, three NETG3, and two MiNEN lines ^(21)^. These models recapitulated key pathohistological aspects, genetic mutation profiles, and drug responses of the corresponding tumors of origin ^(21)^. Notably, none of the three GEP-NETG3 organoids harbored *TP53* or *RB1* alterations, consistent with previous genomic studies distinguishing NETG3 from NEC. Additionally, several independent groups have successfully established NEN organoid models, further supporting the utility of organoid technology as a robust platform for studying NEN biology and therapeutic vulnerabilities ^(49), (50), (51)^.

## Future Prospective

GEP-NEN has recently gained increasing recognition as a disease that requires effective therapeutic intervention. This growing attention is largely attributable to the rising prevalence of the disease and the relatively limited treatment strategies, compared with those for other major cancer types. In this context, 177Lu-DOTATATE has markedly expanded the therapeutic landscape of GEP-NETs. One of the major advantages of 177Lu-DOTATATE is the practical and simple eligibility that can be determined based on the expression of SSTR. Nevertheless, it is important to note that the fundamental biological complexity of this disease yet remains unsolved. As discussed, GEP-NENs represent a highly heterogeneous disease, and their current pathological classification predominantly relies on Ki-67 index. Consequently, there is a gap between the pathological classification system and the biomarkers currently used to guide therapy. Indeed, SSTR expressions are reported not only in GEP-NET but also in GEP-NEC, indicating that SSTR expression cannot distinguish GEP-NEC from GEP-NET ^(52), (53)^. One important challenge is the biological overlap and potential misclassification between GEP-NETG3 and GEP-NEC. The results of the NETTER-2 trial have encouraged the use of 177Lu-DOTATATE in tumors with higher Ki-67 indices. However, it should be noted that the median time to clinical benefit after initiation of 177Lu-DOTATATE has been reported to be approximately 9.7 months ^(54)^. This creates a clinical dilemma, particularly in patients with biologically aggressive disease, in whom treatment decisions must balance the likelihood of response against the time required for therapeutic benefit to emerge. The following case illustrates the clinical challenges associated with this disease. A 56-year-old woman was diagnosed with pancreatic NETG3, with a Ki-67 index of 40%. SRS demonstrated a strong SSTR expression with a Krenning score of 3-4. At diagnosis, the metastatic burden appeared limited, and treatment with 177Lu-DOTATATE was initiated. However, despite receiving two cycles of therapy, her clinical condition deteriorated rapidly, and she passed away 6 months after the diagnosis (Figure 2). This case highlights the difficulty of predicting biological aggressiveness in tumors that fall within the NETG3 spectrum. Although the tumor exhibited favorable characteristics for PRRT, including strong SSTR expression, the clinical course was highly aggressive. These observations suggest that treatment decisions based solely on SSTR expression may be insufficient in such biologically heterogeneous diseases. Particularly, when considering 177Lu-DOTATATE as an early-line treatment, additional biomarkers capable of predicting tumor aggressiveness and disease trajectory are needed to optimize patient selection.

**Figure 2.**
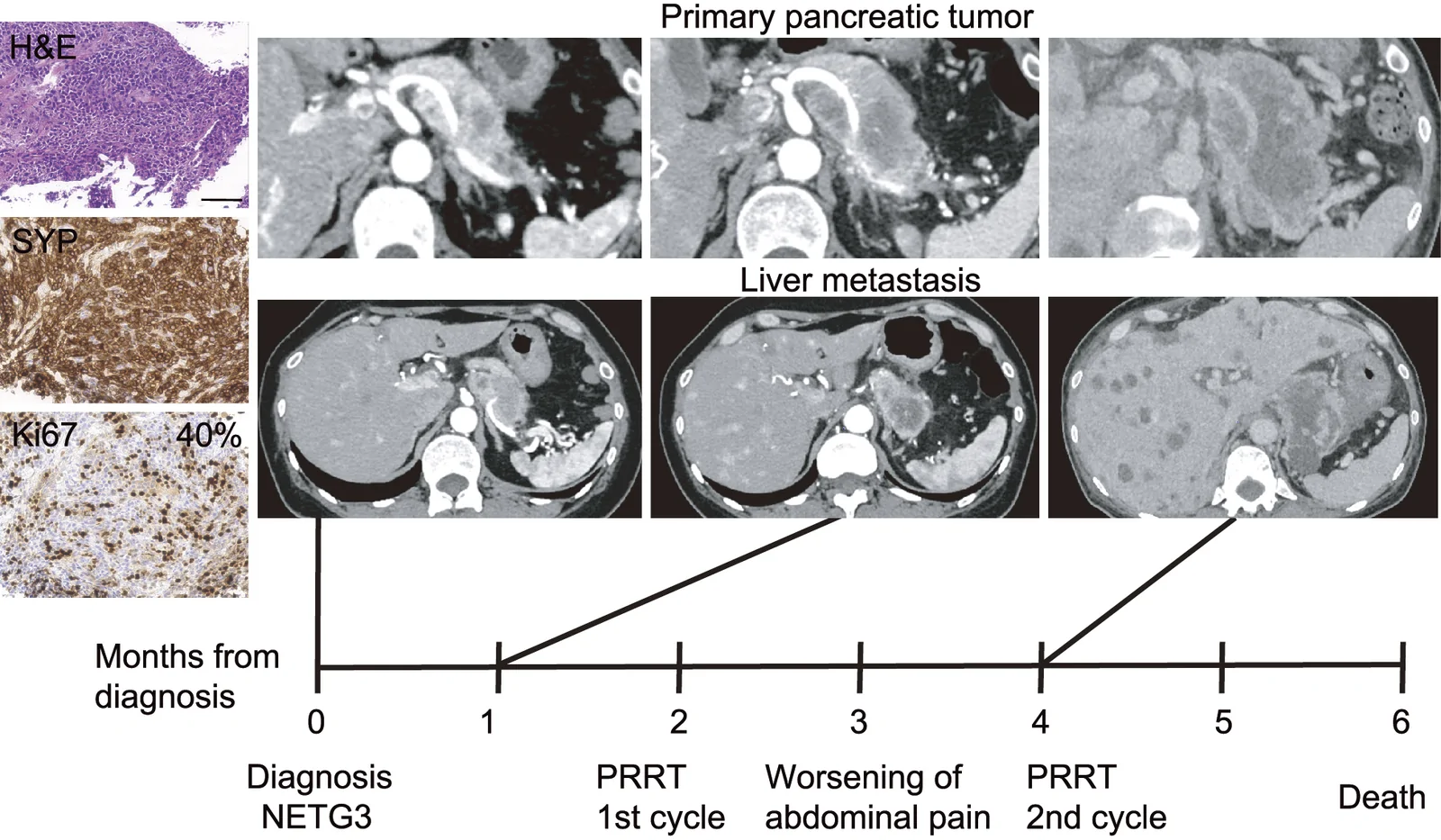
Clinical course of a representative patient with pancreatic NETG3. The patient was diagnosed as NETG3 from the representative pathology (Ki-67 index of 40%). Despite strong SSTR expression and treatment with 177Lu-DOTATATE PRRT, the patient experienced rapid clinical deterioration and passed away within 6 months of diagnosis. Written informed consent was obtained for research use and publication. 177Lu-DOTATATE: Lutetium-177-DOTA-Tyr3-octreotate; H&E: hematoxylin and eosin; NETG3: neuroendocrine tumor G3; PRRT: peptide receptor radionuclide therapy; SSTR: somatostatin receptors; SYP: synaptophysin.

Addressing the biological and clinical heterogeneity of GEP-NENs requires a multifaceted approach integrating clinical and translational research. The application of cutting-edge analytical technologies using patient-derived materials, together with experimental animal models, will be essential for advancing our understanding of this complex disease. Recent studies have begun to apply single-cell ribonucleic acid sequencing and spatial transcriptomic analysis to human NET samples, providing unprecedented insights into tumor heterogeneity and cellular interactions ^(55), (56)^. In parallel, GEP-NEN organoid models have emerged as powerful platforms for investigating oncogenesis, lineage plasticity, metabolism, and therapeutic vulnerabilities in GEP-NENs ^(57)^. These models not only facilitate the discovery of novel therapeutic targets and drug repositioning opportunities but also provide a framework for functional validation of biological hypotheses ^(21), (58)^. In addition, understanding the mechanisms underlying metastatic progression remains a major unmet need in the field ^(59)^. Given that metastatic disease is the primary cause of mortality in patients with GEP-NENs, elucidating the factors that govern metastatic potential may provide new opportunities for both biological discovery and therapeutic intervention.

Our understanding of GEP-NEN biology is still in its early stages. Continued integration of genomic, transcriptomic, spatial, and functional approaches will be necessary to refine disease classification, to improve patient stratification, and ultimately, to develop more effective therapeutic strategies for this heterogeneous group of neoplasms (Figure 3).

**Figure 3.**
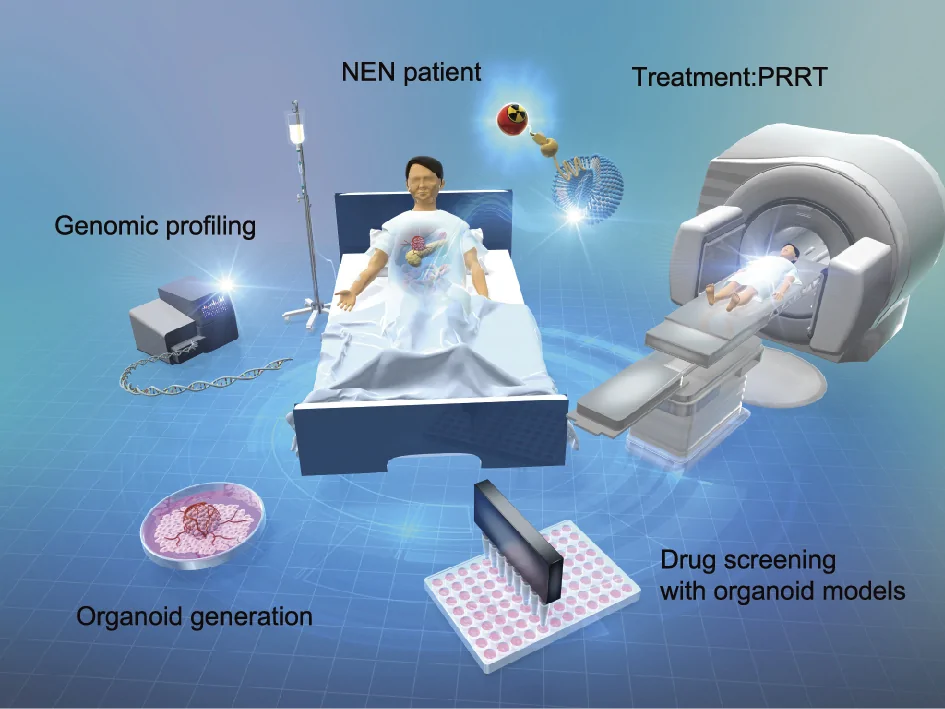
A schematic overview of an integrated clinical and translational approach to GEP-NENs. Comprehensive genomic profiling may facilitate treatment selection, while patient-derived organoid models provide a platform for biological investigation and therapeutic discovery. Together, these approaches may improve patient stratification and optimize the application of PRRT and other treatment strategies. GEP-NEN: gastroenteropancreatic neuroendocrine neoplasm; PRRT: peptide receptor radionuclide therapy.

## Article Information

### Acknowledgments

The author is grateful to the Medical Research Encouragement Prize Committee of the Japan Medical Association in 2025 for selecting our research for this prestigious award.

The author also expresses his deepest appreciation for the patients, colleagues, mentors (especially Drs. Toshiro Sato [Keio University], Joan Massagué [Memorial Sloan Kettering Cancer Center], and Charles M. Rudin [Memorial Sloan Kettering Cancer Center]) who made this research possible. Portions of this manuscript were edited for clarity and grammar using ChatGPT (OpenAI). No content was generated without author verification, and the authors take full responsibility for the final text.

### Conflicts of Interest

None

This article is based on the study, which received the Medical Research Encouragement Prize of the Japan Medical Association in 2025.
